# Recent Applications of Chitin- and Chitosan-Based Polymers in Plants

**DOI:** 10.3390/polym11050839

**Published:** 2019-05-08

**Authors:** Massimo Malerba, Raffaella Cerana

**Affiliations:** 1 Dipartimento di Biotecnologie e Bioscienze, Università degli Studi di Milano-Bicocca, 20126 Milan, Italy; massimo.malerba@unimib.it; 2 Dipartimento di Scienze dell’Ambiente e della Terra, Università degli Studi di Milano-Bicocca, 20126 Milan, Italy

**Keywords:** chitin, chitosan, defense responses, nanoparticles, plant growth, pesticides

## Abstract

In recent years, the use of complex molecules based on the natural biopolymer chitin and/or on its deacetylated derivative chitosan has resulted in great advantages for many users. In particular, industries involved in the production of drugs, cosmetics, biotechnological items, and food have achieved better results using these particular molecules. In plants, chitin- and chitosan-based molecules are largely used as safe and environmental-friendly tools to ameliorate crop productivity and conservation of agronomic commodities. This review summarizes the results of the last two years on the application of chitin- and chitosan-based molecules on plant productivity. The open questions and future perspectives to overcome the present gaps and limitations are also discussed.

## 1. Introduction

Over the past decades, the use of products alternative to pesticides and chemical fertilizers in the control of pre- and post-harvest diseases and in the perspective to increase crop productivity has become more and more important in agriculture. Among the large variety of alternative products proposed so far, shrimp and crab exoskeleton powder chitin (CH) and its deacetylated derivative chitosan (CHT) have been utilized by farmers as biopesticides, biofertilizers and as agricultural film in seeds and fruits coating since the 1980s [1]. Various methods of application of CH and CHT have been proposed (e.g., soil amendment, foliar, seed, and fruit application; CH or CHT alone or in combination with other treatments) to prevent the development of plant diseases or trigger plant innate defenses against pathogens [1]. The obtained results may vary and depend on various parameters including the pathosystem, the applied molecule, its concentration, the degree of deacetylation, viscosity, and the used formulation. To overcome these matters, in recent years many scientists have proposed to test molecules in which CH and CHT have been linked to other chemicals. The utilization of these complex molecules instead of CH and CHT alone has resulted in great advantages for chemical, biotechnological, cosmetic, and food industries [2]. Recent advances in the use of CHT polymers in agriculture were presented in a previous review [2]. Here, we summarize the results obtained during the last two years on the effects of chitin- and chitosan-based molecules supplied by traditional agricultural practices (e.g., seed and foliar spraying, soil amendment) on plant productivity and protection against pathogens with particular focus on nanoparticles. 

## 2. Chitin-Based Polymers

Due to its high nitrogen content and low C/N ratio, CH can be directly used as a fertilizer to enhance crop growth. The addition of chitin to the soil also improves microbial communities in both the abundances and structures. Application of CH in biocontrol of plant pathogens has also been extensively explored. In plants, CH is recognized by specific receptors present on the cell plasma membrane, such as the pathogen-associated molecular pattern (PAMP) receptor. After this recognition, CH can activate PAMP-triggered immunity that is able to induce defense responses against potential fungal, bacterial, and viral pathogens [3]. However, because of its high crystallinity, commercially available bulk CH is not soluble in most common solvents. Despite its utilizable functions, this lack of solubility limits not only its use in basic research, but also its practical application in agriculture [3]. In the search to overcome this limitation, several strategies have been adopted (Table 1).

For example, the use of CH obtained from alternative sources was tested. Nowadays, the major source of this compound is crustacean waste. Sun and coworkers [4] proposed to test CH obtainable from *Saccharomyces cerevisiae* cell wall. CH extracted from yeast and other fungi has several advantages: (i) it cannot cause allergies in humans and animals like CH from arthropods; (ii) it has physical and chemical properties such as biodegradability, biocompatibility and nontoxicity, that make its use in agriculture easier [4]. Postharvest treatment of tomato fruits with CH from a yeast cell wall effectively induced strong resistance against gray mold rot caused by *Botrytis cinerea*. The authors attributed the increased disease resistance of CH-treated tomato fruit to the accumulation of reactive oxygen species, to callose deposition, and to the increase in activities of defense-related enzymes, including superoxide dismutase, catalase, peroxidase, and chitinase along with the increase in the expression of the corresponding genes [4]. Other strategies propose the use of CH with lower molecular weight and therefore more easily soluble. For example, a CH oligosaccharide, obtained by mechanical grinding, autoclaving, and sonication of shrimp shells CH induced in *Arabidopsis thaliana,* the expression of genes related to vegetative growth and carbon and nitrogen metabolism. Compared with untreated plants, plants treated with the CH oligosaccharide showed increased fresh weight (10%), radicle length, and total carbon and nitrogen content [5]. Alternatively, grinder pretreatment and the subsequent use of a high-pressure water jet system can disintegrate CH from crustacean shells into nanofibers. CH nanofibers (CNF) can be more feasibly used since they behave as a water-soluble material. Thus, they were tested for their elicitor capacity, and Egusa et al. first showed that CNF enhanced resistance against pathogens in *A. thaliana* and rice [6]. More recently, when challenged with the fungal pathogens, cabbage and strawberry plants, grown in a mixture of soil and CNF, showed a reduction in the number of spots caused by *Alternaria brassicicola* and lesion size by *Colletotrichum fructicola* in respect to control plants [7]. The addition of CNF to soil slightly enhanced tomato plants growth and reduced the incidence of Fusarium wilt disease relative to CNF-untreated plants. Disease inhibition was more effective utilizing CNF complexed with both protein and CaCO_3_ in protein/CaCO_3_/chitin nanofiber (P/Ca/CNF) or only with protein in protein/chitin nanofiber (P/CNF) [8]. Similarly, acidic hydrolysis of shrimp CH can lead to obtain rod-like particles with a cationic nature and high biological activities named nanochitin whisker (NCW). In outside pot experiments, two varieties of winter wheat treated with the NCW showed enhanced yield with significant increases of net photosynthesis, stomatal conductance, intercellular CO_2_ concentrations, and transpiration rate in flag leaf at the grain filling stage. Grain protein, iron, and zinc contents also increased in NCW-treated wheat [9]. In wheat, the antifungal activity of NCW was also tested. Liang et al. [10] showed that in a pot test NCW had significant inhibitory effects on mycelial growth and conidial production of *Fusarium pseudograminearum* and *F. graminearum* causing agents of crown rot, the most damaging disease in this crop. In indoor and field trials, NCW also stimulated tobacco seed germination and seedling growth (i.e., stem length and girth, leaf number and area). In addition, when mixed with metalaxyl mancozeb and thiophanate methyl fungicides, NCW had synergistic effects on inhibition of tobacco root rot, suggesting its potential utilization for reducing the use of chemical fungicides in tobacco plantations [11]. Finally, CH nanoparticles mechanically prepared from shells of *Penaeus semisulcatus* were fused to iron. In in vitro experiments using standard antibiotics as positive controls, the obtained nanocomposite showed antimicrobial activity against *Pseudomonas aeruginosa, Escherichia coli*, *Salmonella typhi*, and to a lesser extent against *F. oxysporum,* suggesting its utilization against various bacterial and fungal pathogens [12].

## 3. Chitosan-Based Polymers

Extensive use of chemicals in agriculture to increase plant productivity and resistance against pathogens can cause irreversible damage to the ecosystem due to their accumulation in the environment and in the living organisms. In addition, it can induce the appearance of resistance in plant pathogens and the subsequent necessity to introduce new agrochemicals and/or to increase the use dosage of existing products [13]. This obviously increases costs and risks. In the search for new approaches to solve this dilemma, application of nanotechnology appears very promising. Chitosan (CHT) is one of the most useful biomaterials in nanotechnology due to its biodegradability, biocompatibility, and nontoxicity to humans. In addition, CHT can easily be modified without affecting its innate capabilities as compared to other biopolymers like chitin, starch, gelatin, cellulose, and glucans [14]. Therefore, CHT has been widely used for various applications through modifications of its physicochemical and biophysical properties. Recently, in plants, a large spectrum of antimicrobial and regulatory activities were obtained by the use of CHT-based molecules. For example, CHT nanoparticles (CHTNP), having the properties of CHT and the characteristics of nanoparticles such as surface and interface effect, small size and quantum size effects, were able to act as germination elicitor of *Oryza sativa* L. [15] and to positively affect seed germination and seedling growth of wheat (*Triticum aestivum* L.) [16]. In addition, these nanoparticles were able to induce the expression of pathogenesis-related proteins thus enhancing resistance against *F. andiyazi* in tomato [17] and to inhibit *F. oxysporum* growth in *Zingiber officinale* [18]. CHTNP complexed with other molecules were largely used to promote plant growth and to induce resistance against phytopathogens. Entrapment of nitrogen, phosphorous, and potassium into CHTNP enhanced uptake of nutrients, photosynthesis, and growth of treated coffee plants [19] and induced starch and major proteins accumulation in *Pisum sativum* plants [20]. CHTNP complexed with different metals were tested for growth promotion and induction of resistance (Table 2). 

Foliar and seeds treatment with zinc-loaded CHTNP enhanced gluten content in durum wheat [21] and promoted crop yield in maize [22], while Cu- and Zn-CHTNP suppressed cotton seedling disease caused by *Rhizoctonia solani* [23]. Cu-CHTNP increased yield, plant growth, and nutrient content of onion bulbs [24], and it stimulated growth and induced resistance in finger millet plants against blast disease caused by *Pyricula grisea* [25]. Different metal- CHTNP (CuO-, ZnO, and Ag-CHTNP) prevented Fusarium wilt, the seed borne and soil borne disease caused by *F. oxysporum* on chickpea [26]. Ag-CHTNP application controlled leaf fall disease caused by *Corynespora cassiicola* on rubber trees [27] and significantly affected *Phytophthora capsici* growth on pepper plants [28], while lanthanum-CHTNP effectively promoted growth and improved disease resistance of rice [29]. 

The use of CHTNP complexed with molecules such as elicitors, secondary metabolites, and phytoregulators was also tested (Table 3). 

Treatment of tomato plants with harpin-CHTNP resulted in different expression of several genes involved in defense response and decreased *R. solani* infection [30]. At the appropriate concentration, tripolyphosphate-CHTNP stimulated micropropagation plant growth on *Capsicum annuum* while toxic doses dramatically caused cessation of plant growth [31]. Thiamine-CHTNP stimulated growth and induced resistance against *F. oxysporum* in chickpea seedlings [32], while salicylic acid-CHTNP positively modulated plant growth and improved defense responses on lettuce seedlings [33] and promoted growth and defense in maize [34]. Under laboratory conditions, CHTNP, encapsulated with *Cymbopogon martinii* essential oil, exhibited antifungal and antimycotoxin activities against *F. graminearum* in maize grains [35]. Coumarin-functionalized CHT derivatives affected *A. solani*, *F. oxysporum,* and *F. moniliforme* growth [36]. CHT, vanillin, and salicylic acid hybrids effectively induced resistance against *Puccinia recondita* and *Cochliobolus sativus* in wheat [37]. A bentonite-CHT nanoclay showed antimicrobial action against *P. syringe* and *F. solani* and exerted elicitor action on tomato plantlets [38]. CHTNP derivatives positively affect the responses to abiotic stresses, too. For example, Cu-CHTNP enhanced growth and expression of defense genes in tomato plants under salt stress [39], and *S*-nitrosoglutathione-CHTNP improved drought tolerance of sugarcane plants [40]. 

Finally, CHT-based polymers were also tested for the construction of film for fruits and seeds coating (Table 4).

Application of CHTNP extended shelf life and maintained quality of banana fruits [41], while CHT + procyanidins coating promoted quality maintenance and inhibited microbial growth of fresh blueberry [42]. Coating with *Ficus hirta* Vahl. fruits extract-incorporated CHT reduced postharvest loss and enhanced the storability of Xinyu tangerines during cold storage by antimicrobial activity [43]. Coating with a photoactivated chlorophyllin-CHT conjugate diminished microbial contamination thus prolonging the shelf life of strawberries without any negative impact on commodities aspect and quality [44]. Treatment of winter jujube fruits with a CHT/nano-silica/sodium alginate composite film prolonged the fruit shelf life by increasing the expression of antioxidant genes [45]. In addition, CHT/nano-TiO_2_ and chitosan/nano-SiO_2_ coating films preserved *Ginco biloba* seeds quality by positively affecting the activities of scavenger antioxidant enzymes [46].

## 4. Open Questions, Future Perspectives, and Conclusions

In plants, investigations of the molecular mechanism of CH perception and CH-induced immunity led to the discovery of a specific receptor, which is a plasma membrane LysM- containing protein. After the recognition of CH by this receptor, a well-defined signaling cascade leads to the responses [47]. Different mechanism of action of CHT and its derivatives are reported in the literature. Metal-chelation and/or interaction between positively charged CHT and anionic molecules of cell/wall/DNA of microbes may lead to leakage of intracellular components or to inhibition of mRNA and protein synthesis. This can directly kill the pathogen. Similarly, CHT appears to elicit responses from plant cells via electrostatic interactions with negatively charged molecules instead of via interaction with a specific receptor like CH [3]. In fact, a strong electrostatic interaction exists between polycationic CHT and polyanionic structures like lipopolysaccharides, proteins, and metal ions present in the cell wall and in the cell plasma membrane. In addition, the negatively charged phosphate groups of DNA offer an excellent binding surface to cationic groups of CHT. This binding can lead to specific modifications in protein expression [48]. Anyway, with a genetic approach some studies suggest the presence of putative CHT receptors [49] and, recently, the CHT perception by the CH receptor CERK1 has been clearly demonstrated [50].

Although there are many reports of the application of CH, CHT, and CHT derivatives in agriculture, much work remains to be done. Most of these studies are still at the laboratory or greenhouse level and field trials are needed. In particular, the effect of these molecules on ambient and soil biome must be evaluated. For example, several reports show that metals- and metal oxides-containing CHT nanoparticles are under advanced investigation for use in agriculture. The reports on the toxicity of these metal-containing CHT derivatives on plants and subsequently on soil, terrestrial ecosystems and humans are contradictory and initial risk studies seem to undervalue toxicity issues. However, recent investigations raised serious worries about the toxicity of residual metals. Thus, the issue of toxicity of these metal-containing compounds in living organisms and their fate in soil, water and air is a source of serious apprehension and several scientists suggest further research for concrete conclusions [2].

A nanotechnological approach in agriculture is very promising to increase the efficacy of agrochemicals and lower their environmental impact. Several evidences show that CHT nanoparticles could be a smart system for the controlled release of various agrochemicals including pesticides, micronutrients, fertilizers, and plant hormones, and they are able to play a dual role in plant growth regulation and protection against phytopathogens [2]. Several commercial products have been developed for application in agriculture, for example CHITOSAN 6 from RUMEXO Ltd (Derby, United Kingdom) and different types of CHT preparations from G.T.C. Bio Corporation (Guingdao, China). In spite of this, the reported applications are limited. The main obstacle that hinders wide utilization of these CHT derivatives in field is the immense and still not well-known bioactivity of these compounds against bacteria, fungi, viruses, and plants. Concomitantly, mechanisms of interaction of CHT derivatives towards microbes and plants are not well understood especially because the different biophysicochemical characteristics of different CHT preparations led to a discrepancy in degree of bioactivities/reproducibility of results [3]. Therefore, since CHT is indeed cumbersome and very expensive to customize into stable and desired nanoforms, the future task for scientists is to implement new large-scale production processes for preparation of the desired CHT-based molecules.

Finally, promising results have been very recently obtained in the plant genetic engineering field. Genetic engineering is an important tool for sustainable increase of crop productivity and biosynthesis of useful molecules. The use of CHT-based molecules seems to allow the overcoming of some concerns about conventional engineering methods like polyethylene glycol-mediated transformation or particle bombardment. In particular, chitosan-complexed single-walled carbon nanotube carriers appeared and are able to deliver plasmid DNA selectively to chloroplasts of different plant species, including tobacco, spinach, and the model plant *A. thaliana* without the risk to affect nuclear genome [51].

To conclude, despite the open challenges, molecules based on CH and CHT have proved useful in various aspects of plant biology, ranging from the increase in crop yield to the protection against the attack of pathogens. The huge number of papers recently published testifies to the interest of scientists for the use in agriculture of these molecules and this should lead to relevant progress in a short period and extension of their use to other parts of plant life.

## Figures and Tables

**Table 1:** Recent application of chitin-based polymers.

Plant Species	Chitin Characteristics	Chitin Effect	Reference
*Solanum lycopersicum*	From *Saccharomyces cerevisiae* cell wall	Resistance against *Botrytis cinerea*	[4]
*Arabidopsis thaliana*	CH oligomers (mainly tetramers)	Increased fresh weight, radicle length and total carbon and nitrogen content	[5]
*Arabidopsis thaliana, Oryza sativa japonica*	CH oligomers	Resistance against *Alternaria brassicicola* and *Pseudomonas syringae*	[6]
*Brassica oleracea*, *Fragaria* x *ananassa*	CH nanofibers	Resistance against *Alternaria brassicicola* and *Colletotrichum fructicola*	[7]
*Solanum lycopersicum*	Protein/CaCO_3_/CH nanofibers or protein/CH nanofibers	Plant growth, resistance against *Fusarium oxysporum*	[8]
*Triticun aestivum*	Nanochitin whisker	Yield, grain protein, iron, and zinc contents	[9]
*Triticun aestivum*	Nanochitin whisker	Resistance against *Fusarium pseudograminearum* and *Fusarium graminearum*	[10]
*Nicotiana tabacum* L.	Nanochitin whisker	Increased seed germination and growth, resistance against *Fusarium* spp.	[11]
*In vitro* test of pathogens growth	Iron/CH nanoparticles	Inhibition of *Pseudomonas aeruginosa*, *Escherichia coli*, *Salmonella typhi* and *Fusarium oxysporum* growth	[12]

**Table 2:** Recent application of chitosan-based nanoparticles complexed with metals.

Plant Species	Metal Complexed to CHT Nanoparticles	Effect	Reference
*Triticum turgidum* var. durum	Zn	Gluten content increase	[21]
*Zea mays*	Zn	Crop yield promotion	[22]
*Gossypium* spp.	Zn or Cu	Resistance against *Rhizoctonia solani*	[23]
*Allium cepa* L.	Cu	Increase of plant growth and nutrient content of bulbs	[24]
*Eleusine coracana*	Cu	Increase of plant growth and resistance against *Pyricola grisea*	[25]
*Cicer arietinum* L.	CuO, ZnO or Ag	Resistance against *Fusarium oxysporum*	[26]
*Hevea brasiliensis*	Ag	Resistance against *Corynespora cassiicola*	[27]
*Piper nigrum*	Ag	Resistance against *Phytophthora capsici*	[28]
*Oryza sativa* L.	La	Growth promotion and improved disease resistance	[29]

**Table 3:** Recent application of CHT-based polymers and nanoparticles complexed with different molecules.

Plant Species	Molecule Complexed to CHT	Effect	Reference
*Solanum lycopersicum*	Harpin	Resistance against *Rhizoctonia solani*	[30]
*Capsicum annuum*	Tripolyphosphate	Stimulation of plant growth and biomass accumulation	[31]
*Cicer arietinum* L.	Thiamine	Stimulation of plant growth and resistance against *Fusarium oxysporum*	[32]
*Lactuca sativa* L.	Salicylic acid	Stimulation of plant growth and improved disease resistance	[33]
*Zea mays* L.	Salicylic acid	Stimulation of plant growth and improved disease resistance	[34]
*Zea mays* L.	*Cymbopogon martinii* essential oil	Resistance against *Fusarium graminearum*	[35]
*Hevea brasiliensis*	Coumarin	Resistance against *Alternaria solanii* and *Fusarium* spp.	[36]
*Triticum aestivum* L.	Vanillin and salicylic acid	Resistance against *Puccinia recondita* and *Cochliobolus sativus*	[37]
*Solanum lycopersicum*	Bentonite	Resistance against *Pseudomonas syringe* and *Fusarium solani*	[38]
*Solanum lycopersicum*	Cu	Stimulation of plant growth and expression of defense genes	[39]
*Saccarum* spp.	*S*-nitrosoglutathione	Improved drought tolerance	[40]

**Table 4:** Recent application of chitosan-based polymers and nanoparticles for fruits and seeds coating.

Plant Species	CHT-Based Polymer	Effect	Reference
*Musa acuminata* AAA group	CHT nanoparticles	Shelf life extension and maintenance of fruit quality	[41]
*Vaccinium myrtillus*	CHT + procyanidins	Promotion of quality maintenance and inhibition of fruit pathogens growth	[42]
*Citrus reticulata* Blanco	CHT + *Ficus hirta* Vahl. fruits extract	Reduction postharvest loss and enhancement of fruits storability	[43]
*Fragaria × ananassa* Duch.	CHT + photoactivated chlorophyllin	Extension of fruit shelf life and inhibition of pathogens growth	[44]
*Zizyphus jujuba* Mill. cv Dongzao	CHT/nano-silica/sodium alginate	Extension of fruit shelf life and inhibition of pathogens growth	[45]
*Ginkgo biloba* L.	CHT/nano-TiO_2_ and CHT/nano-SiO_2_	Preservation of seeds quality	[46]
